# Supplementary figures and images for: Vaginal Microbiome Dynamics of Cows in Different Parities
Source: Animals (Basel). 2023 Sep 10;13(18):2880. doi: 10.3390/ani13182880 (PMC10525485; doi:10.3390/ani13182880)

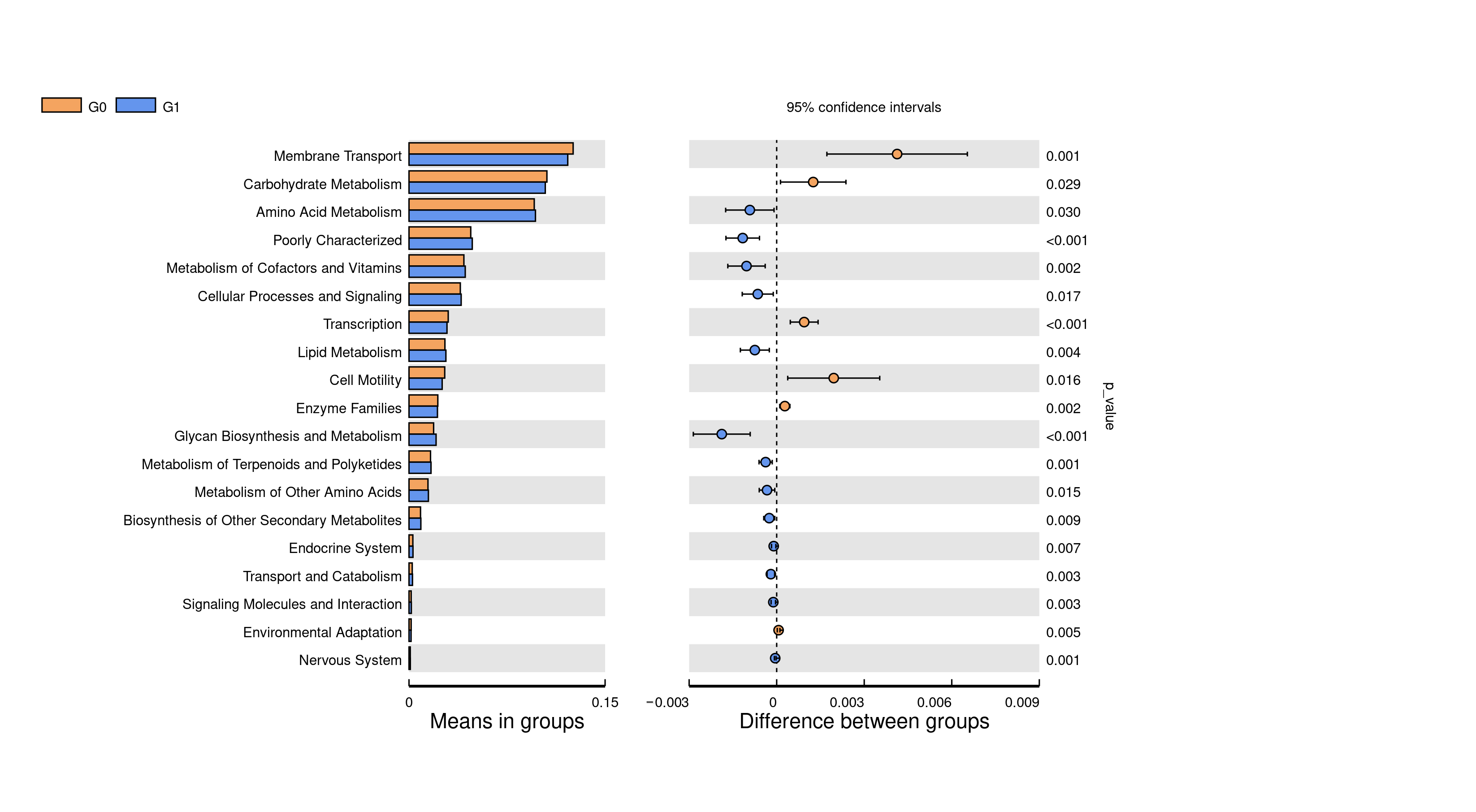

Supplement: Supplementary file 1 [file animals-13-02880-s001.zip › animals-2490486-supplementary/File S1/PICRUSt_ttest_all.fna_group.list_G0vsG1_t_0.05_level2.png]

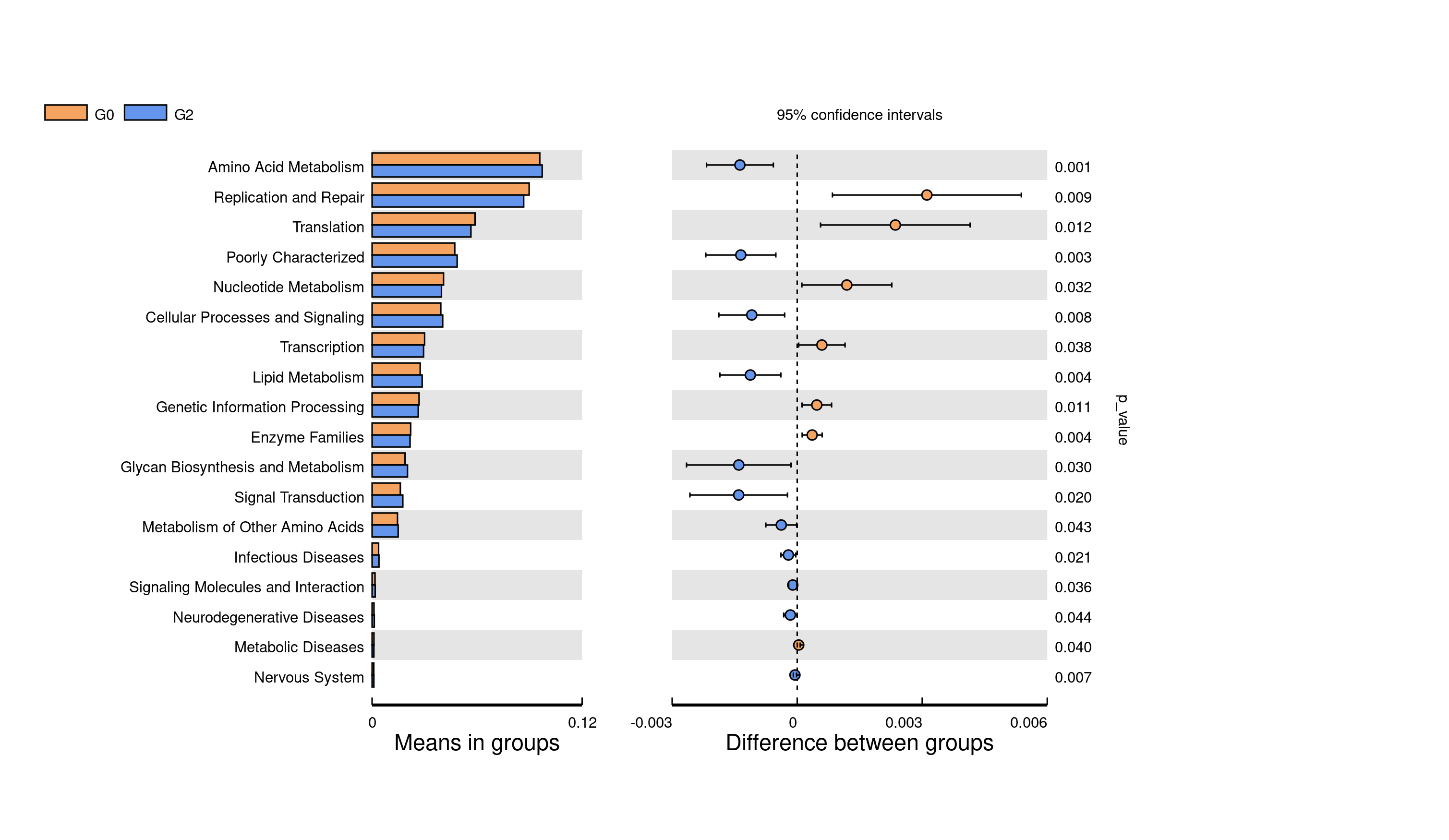

Supplement: Supplementary file 1 [file animals-13-02880-s001.zip › animals-2490486-supplementary/File S1/PICRUSt_ttest_all.fna_group.list_G0vsG2_t_0.05_level2.png]

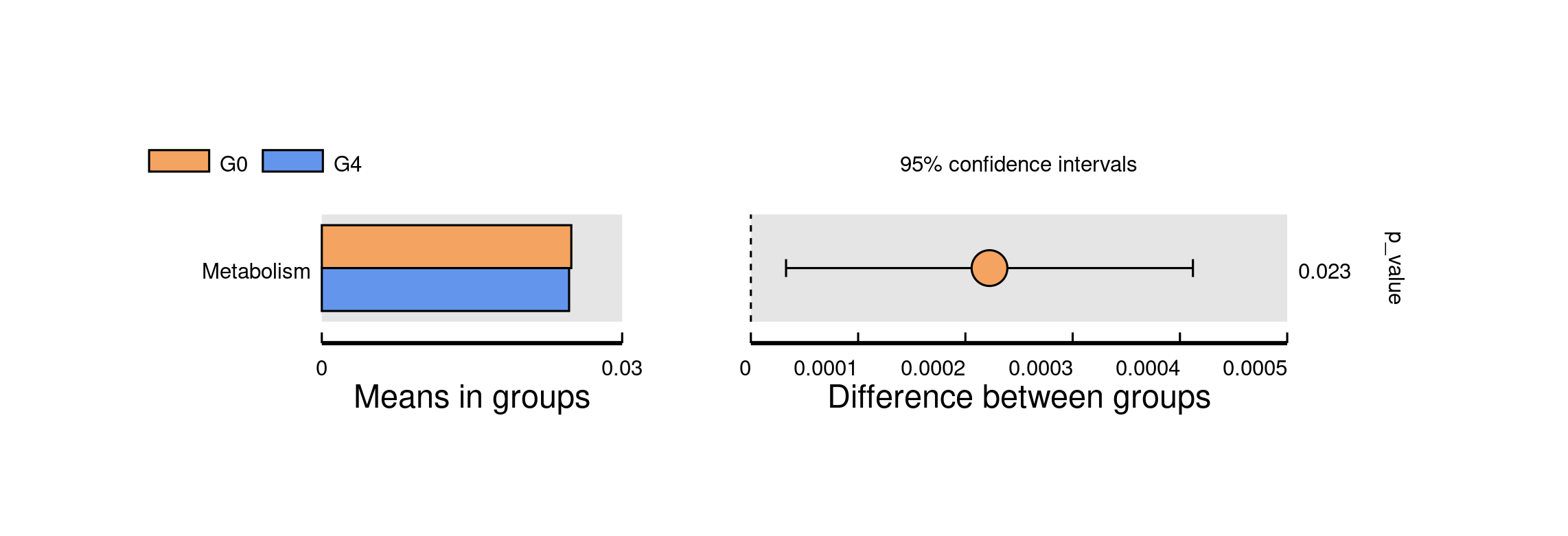

Supplement: Supplementary file 1 [file animals-13-02880-s001.zip › animals-2490486-supplementary/File S1/PICRUSt_ttest_all.fna_group.list_G0vsG4_t_0.05_level2.png]

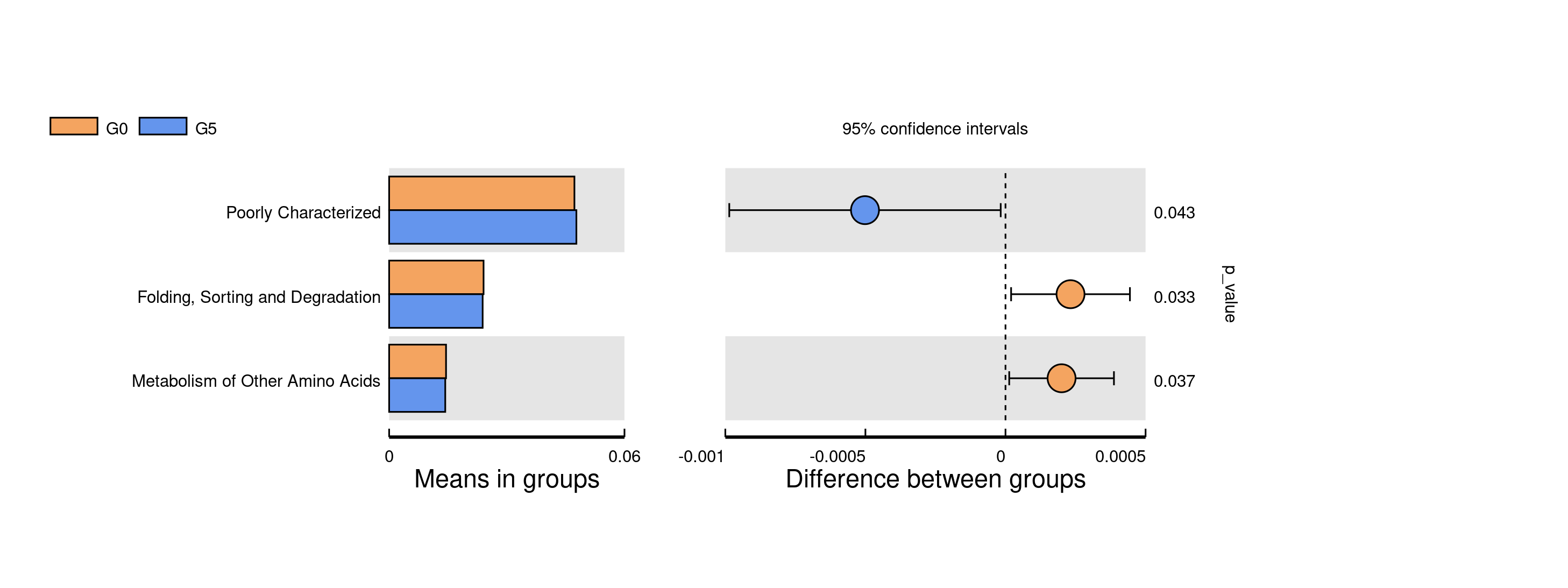

Supplement: Supplementary file 1 [file animals-13-02880-s001.zip › animals-2490486-supplementary/File S1/PICRUSt_ttest_all.fna_group.list_G0vsG5_t_0.05_level2.png]

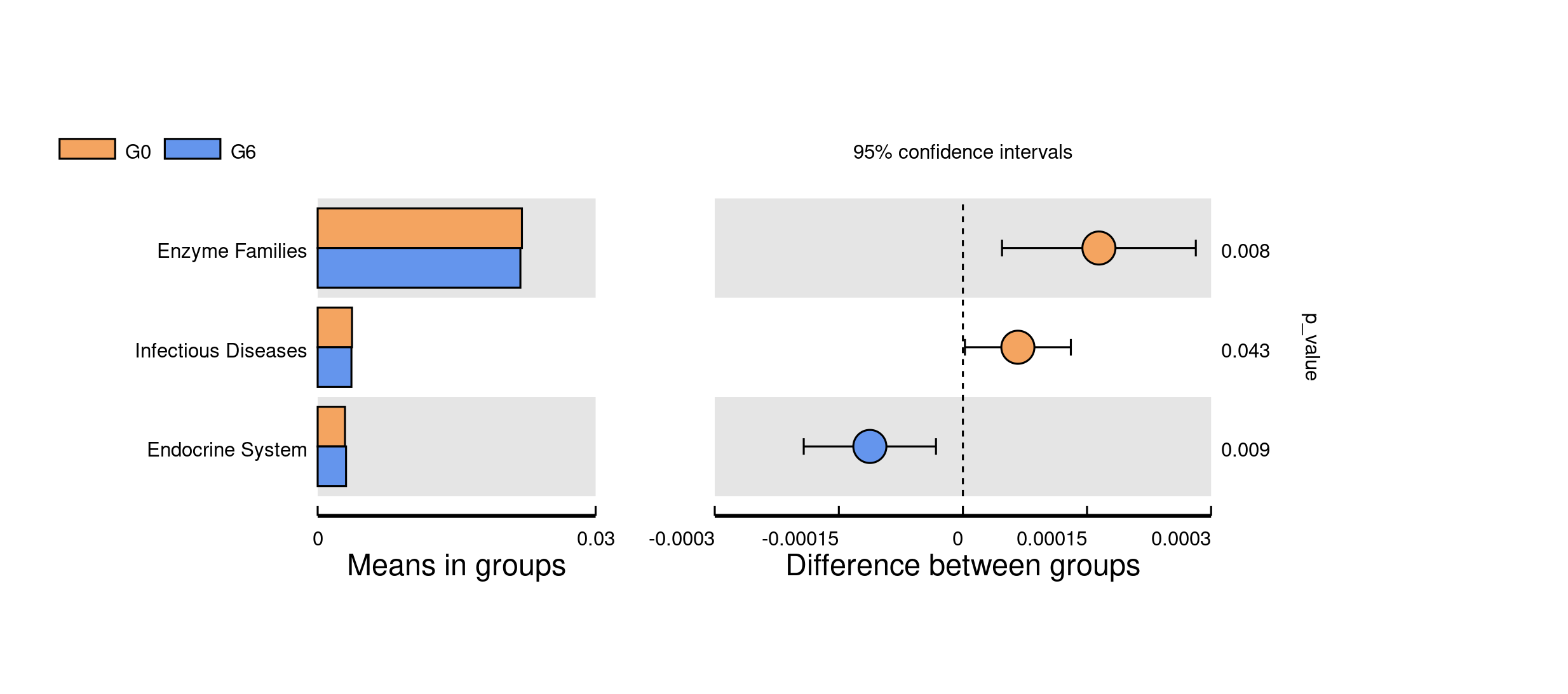

Supplement: Supplementary file 1 [file animals-13-02880-s001.zip › animals-2490486-supplementary/File S1/PICRUSt_ttest_all.fna_group.list_G0vsG6_t_0.05_level2.png]

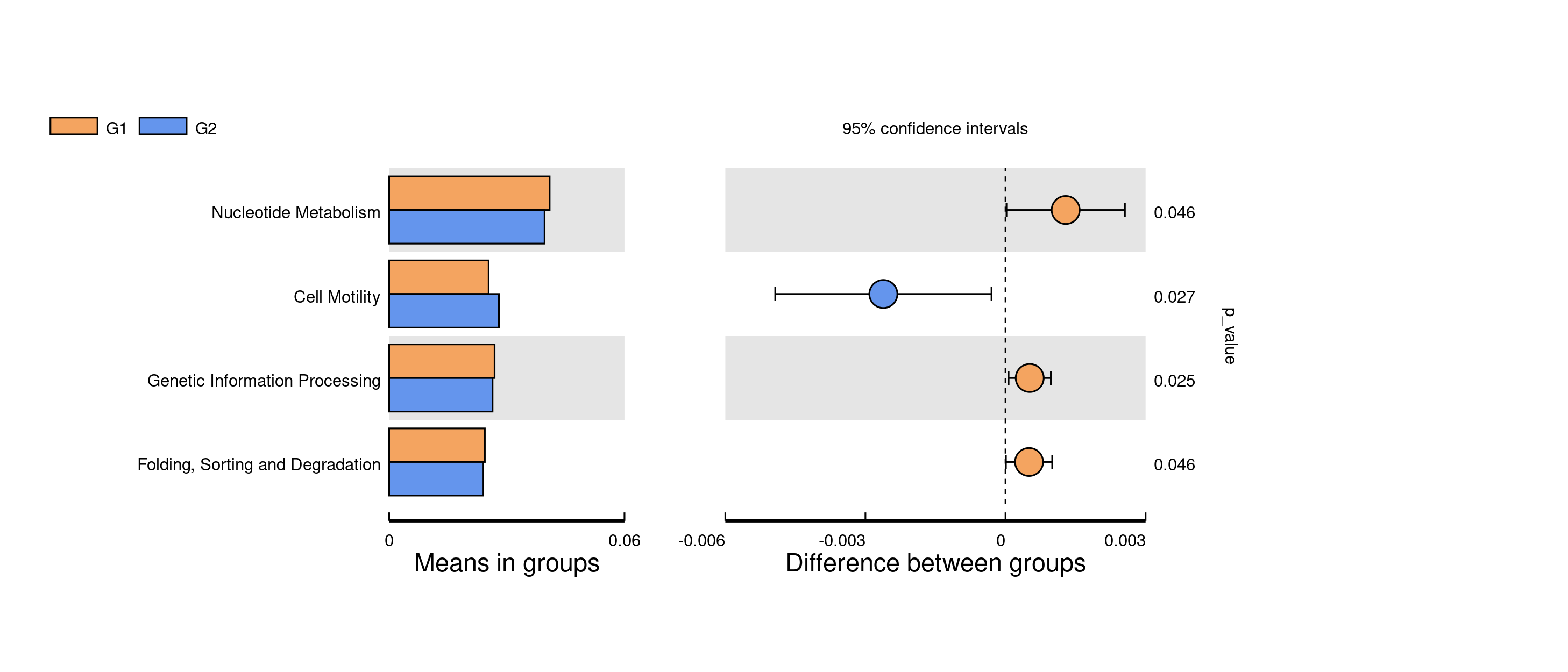

Supplement: Supplementary file 1 [file animals-13-02880-s001.zip › animals-2490486-supplementary/File S1/PICRUSt_ttest_all.fna_group.list_G1vsG2_t_0.05_level2.png]

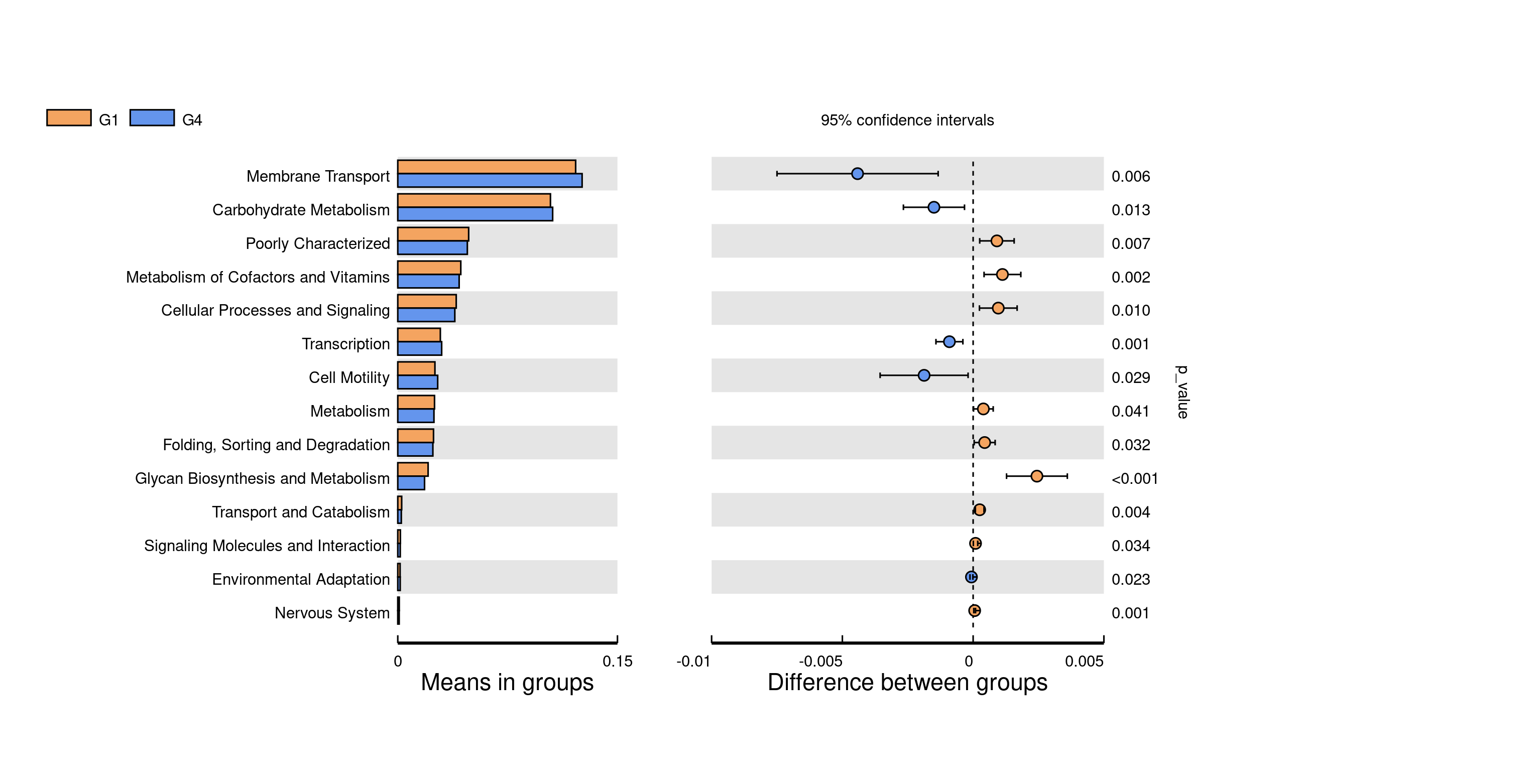

Supplement: Supplementary file 1 [file animals-13-02880-s001.zip › animals-2490486-supplementary/File S1/PICRUSt_ttest_all.fna_group.list_G1vsG4_t_0.05_level2.png]

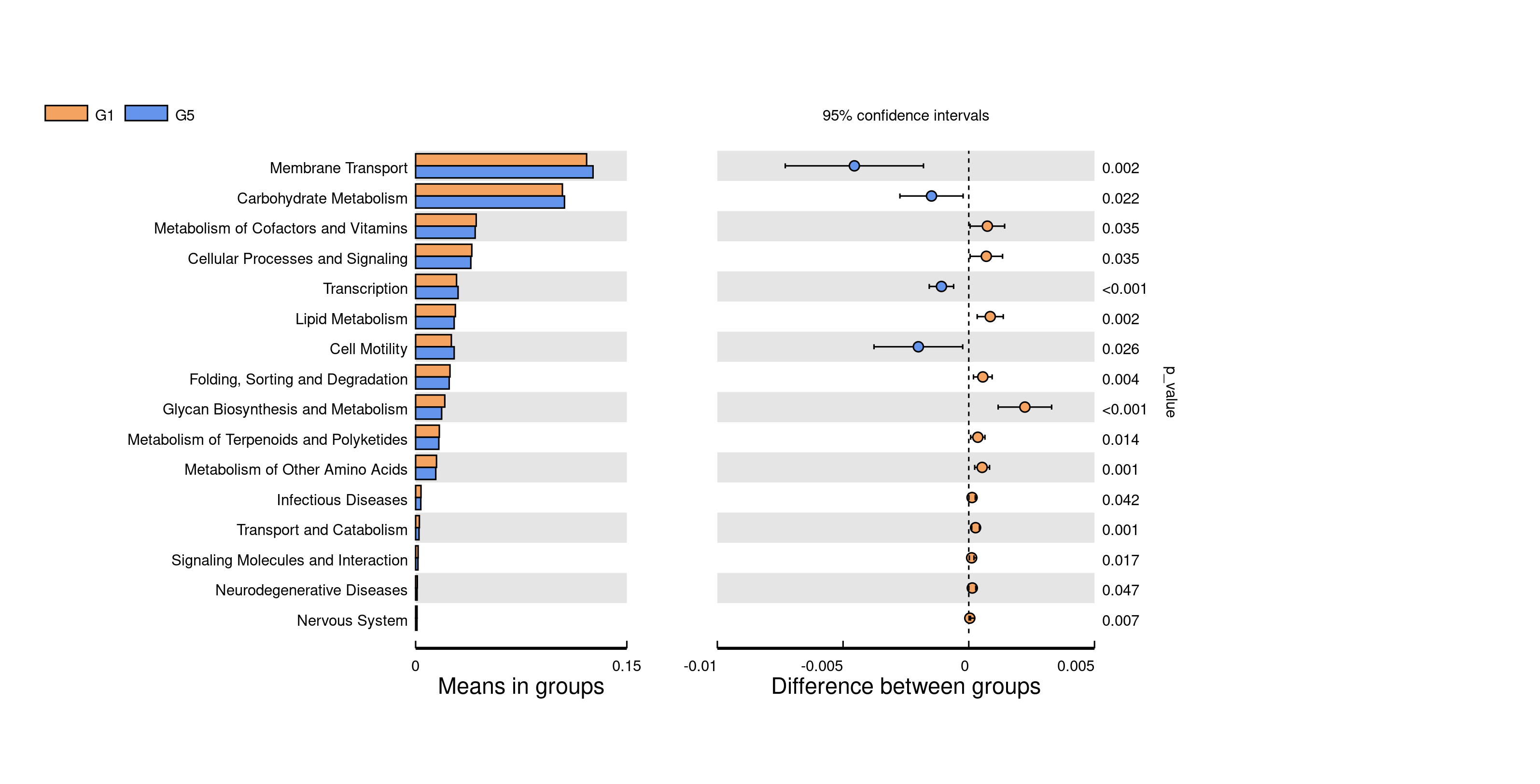

Supplement: Supplementary file 1 [file animals-13-02880-s001.zip › animals-2490486-supplementary/File S1/PICRUSt_ttest_all.fna_group.list_G1vsG5_t_0.05_level2.png]

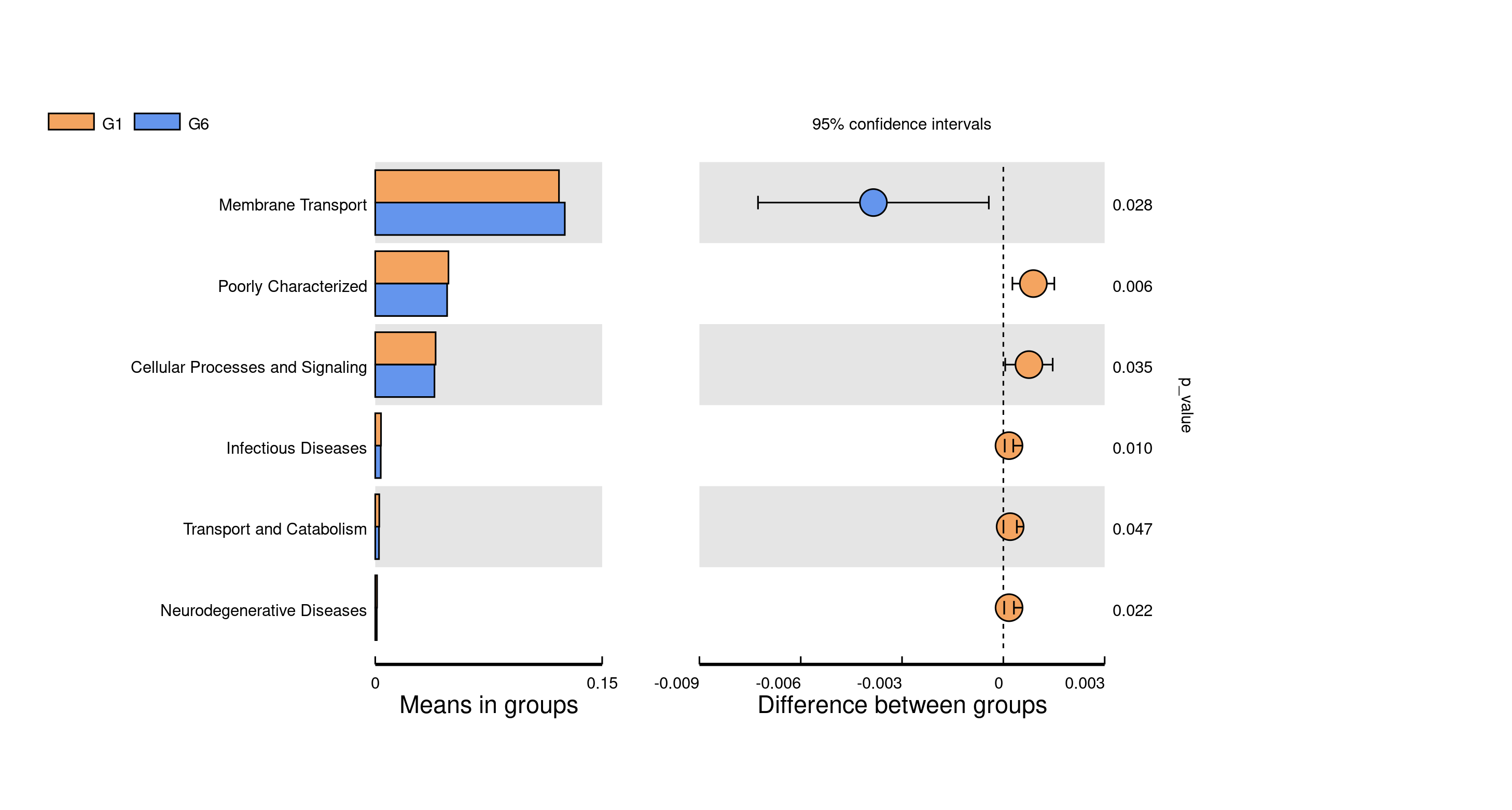

Supplement: Supplementary file 1 [file animals-13-02880-s001.zip › animals-2490486-supplementary/File S1/PICRUSt_ttest_all.fna_group.list_G1vsG6_t_0.05_level2.png]

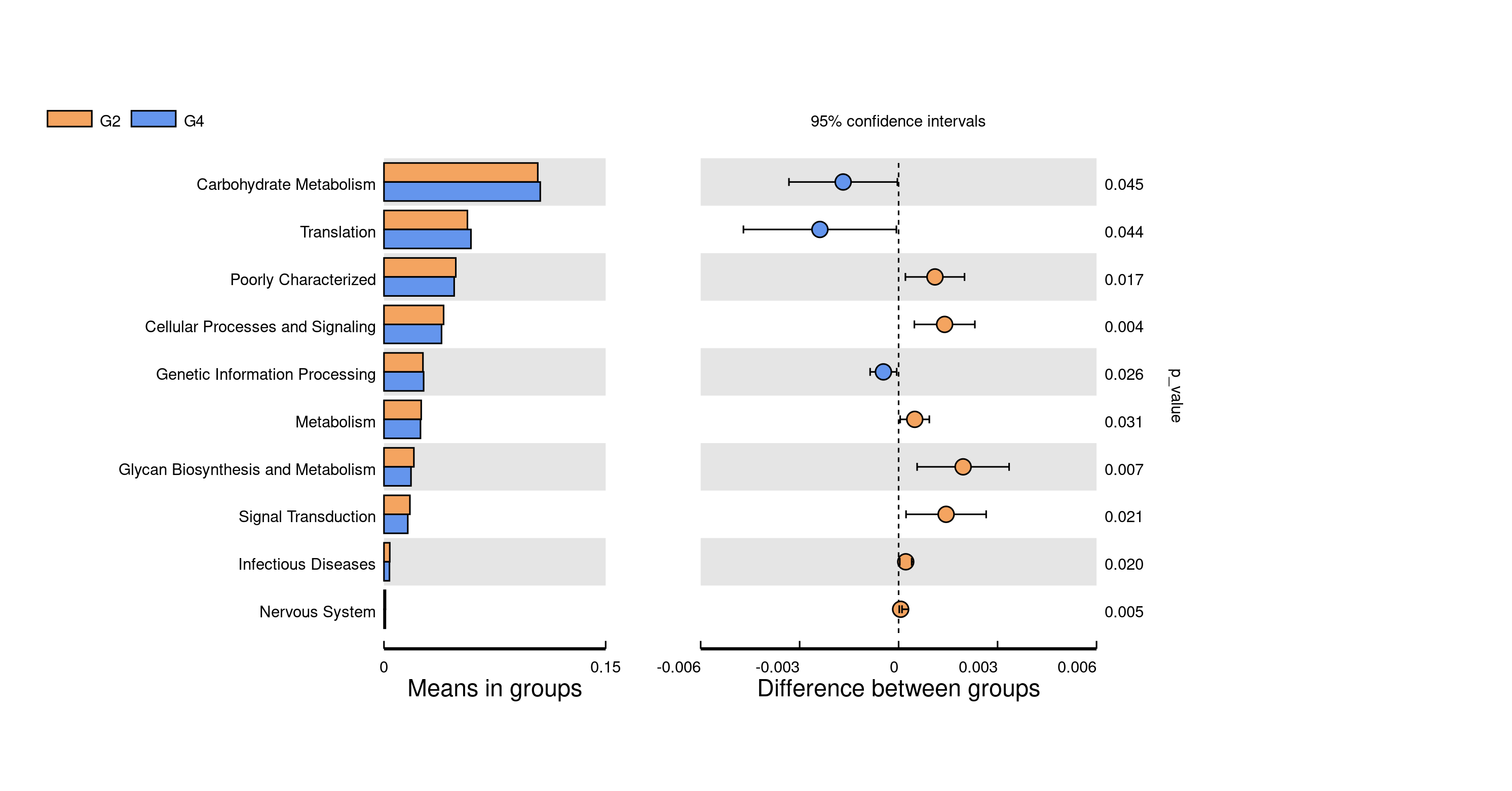

Supplement: Supplementary file 1 [file animals-13-02880-s001.zip › animals-2490486-supplementary/File S1/PICRUSt_ttest_all.fna_group.list_G2vsG4_t_0.05_level2.png]

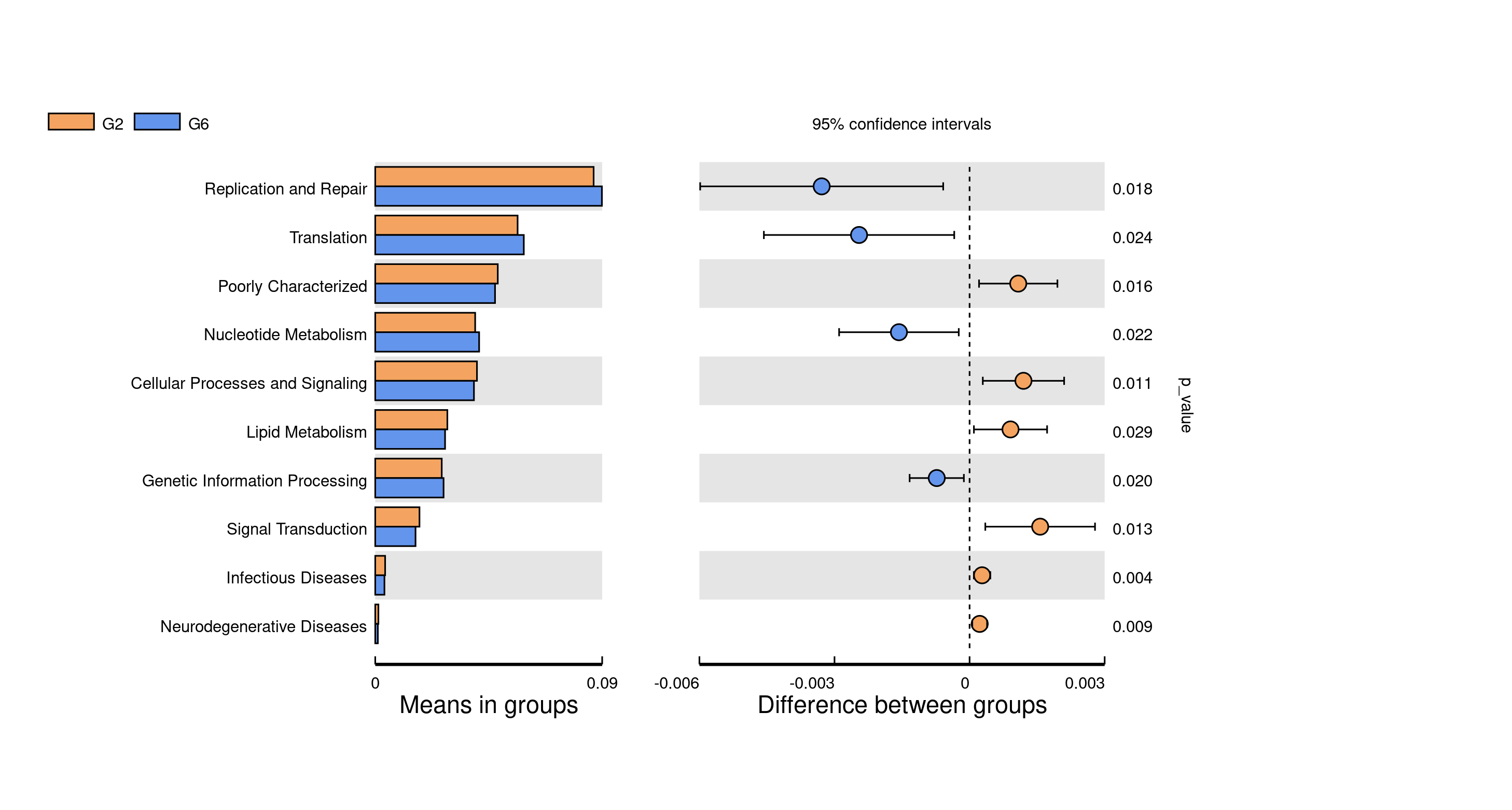

Supplement: Supplementary file 1 [file animals-13-02880-s001.zip › animals-2490486-supplementary/File S1/PICRUSt_ttest_all.fna_group.list_G2vsG6_t_0.05_level2.png]

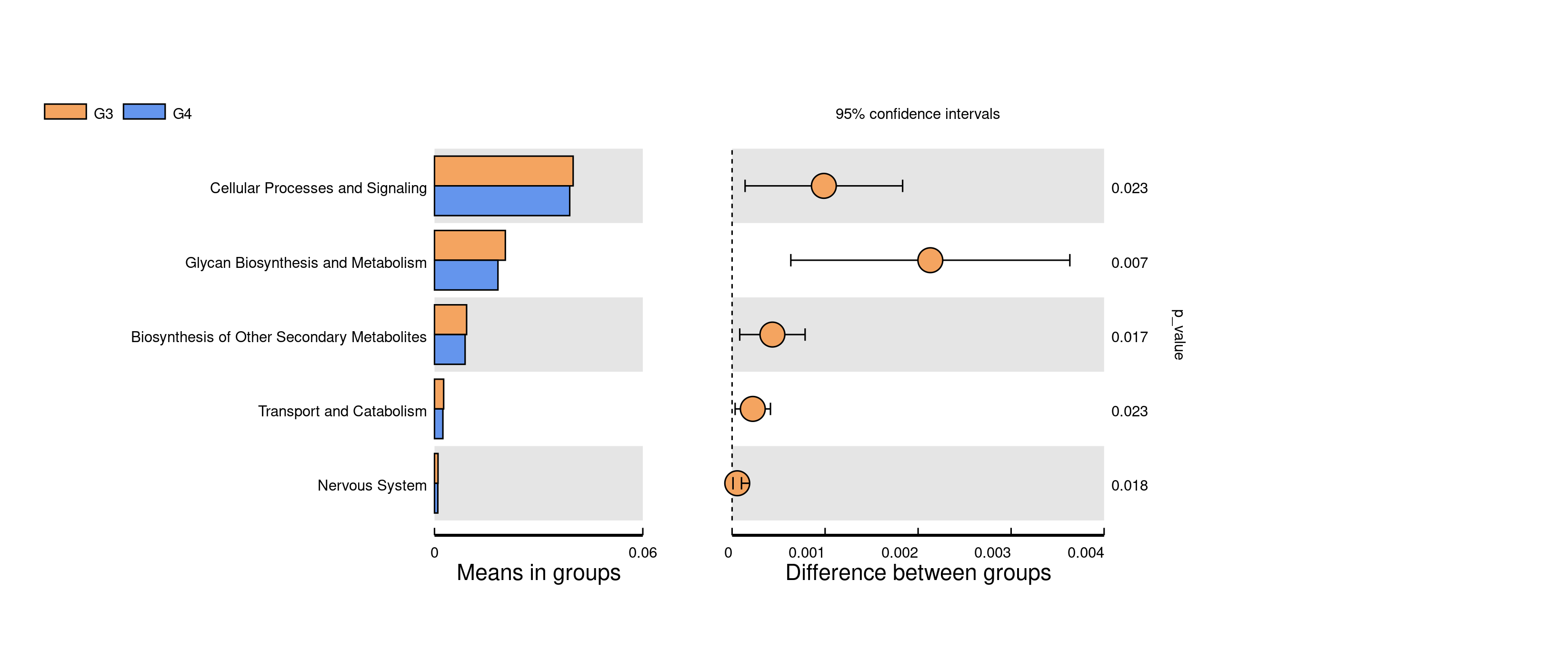

Supplement: Supplementary file 1 [file animals-13-02880-s001.zip › animals-2490486-supplementary/File S1/PICRUSt_ttest_all.fna_group.list_G3vsG4_t_0.05_level2.png]

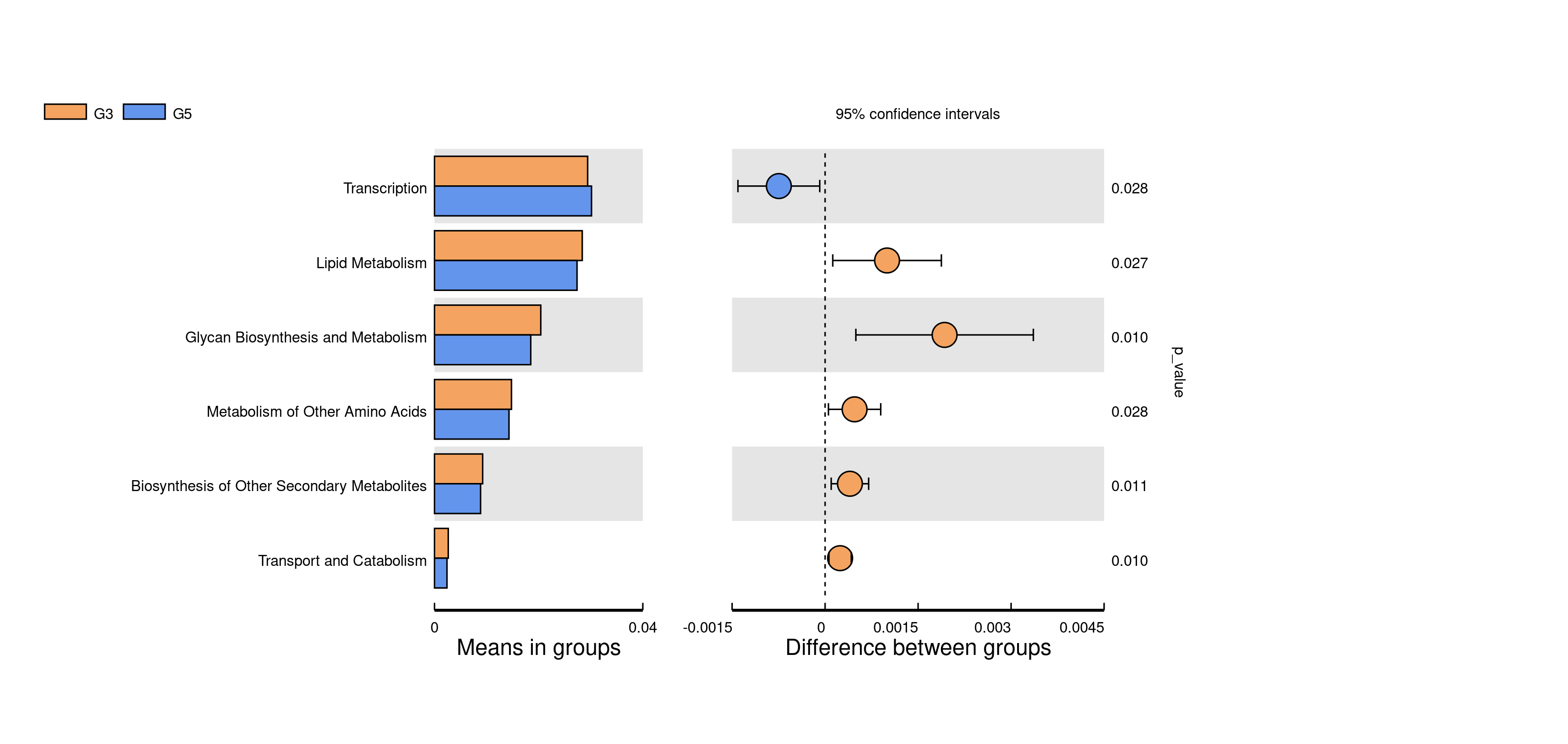

Supplement: Supplementary file 1 [file animals-13-02880-s001.zip › animals-2490486-supplementary/File S1/PICRUSt_ttest_all.fna_group.list_G3vsG5_t_0.05_level2.png]

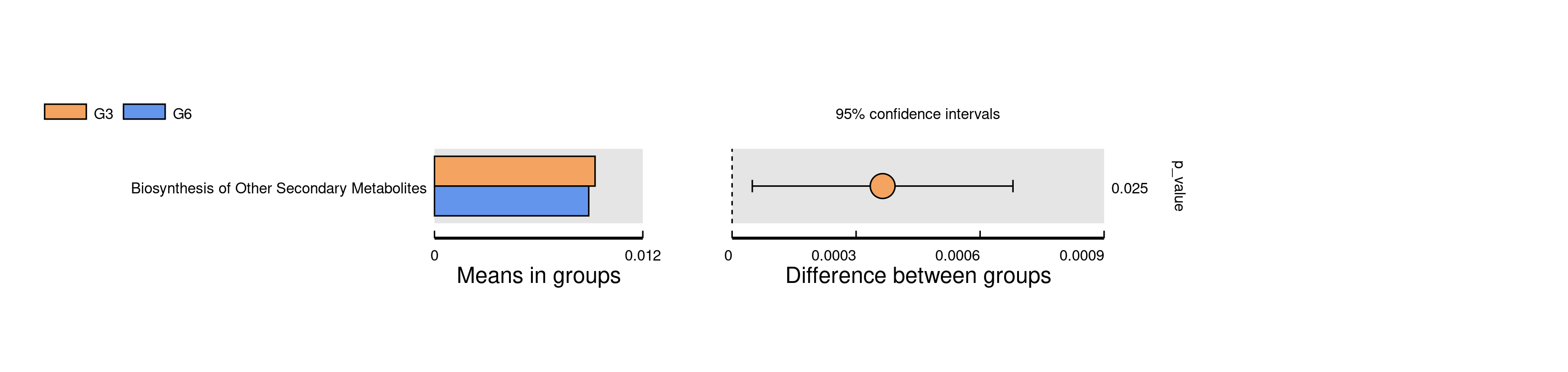

Supplement: Supplementary file 1 [file animals-13-02880-s001.zip › animals-2490486-supplementary/File S1/PICRUSt_ttest_all.fna_group.list_G3vsG6_t_0.05_level2.png]

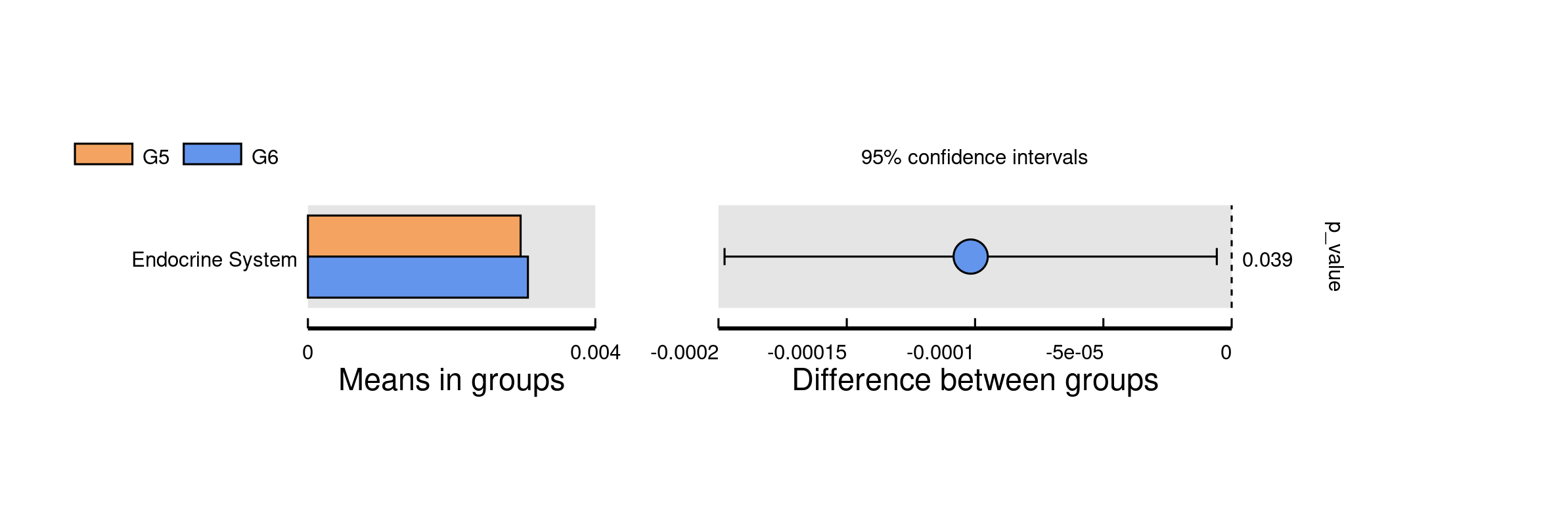

Supplement: Supplementary file 1 [file animals-13-02880-s001.zip › animals-2490486-supplementary/File S1/PICRUSt_ttest_all.fna_group.list_G5vsG6_t_0.05_level2.png]
